# Factors Associated With the Development of Heart Failure Following Acute Coronary Syndrome: A Systematic Review and Meta-Analysis

**DOI:** 10.7759/cureus.75999

**Published:** 2024-12-19

**Authors:** Karanam Satya Sai Venkata Jagadeesh, Tanveer Ahmad Shaik, Abshiro H Mayow, Sindhuja Sompalli, Muhammad Arsalan, Sandipkumar S Chaudhari, Ihtisham Habib, Neelum Ali

**Affiliations:** 1 Internal Medicine, Gomel State Medical University, Gomel, BLR; 2 Cardiovascular Medicine, University of Louisville School of Medicine, Louisville, USA; 3 College of Medicine, St. George's University School of Medicine, St. George, GRD; 4 Internal Medicine, Jagadguru Sri Shivarathreeshwara (JSS) Medical College, Hyderabad, IND; 5 Internal Medicine, Medical Teaching Institute, Lady Reading Hospital Peshawar, Peshawar, PAK; 6 Cardiothoracic Surgery, University of Alabama at Birmingham, Birmingham, USA; 7 Family Medicine, University of North Dakota School of Medicine and Health Sciences, Fargo, USA; 8 Internal Medicine, University of Health Sciences, Lahore, PAK

**Keywords:** acute coronary syndrome, heart failure, predictors, risk factors, systematic review and meta-analysis

## Abstract

Acute coronary syndrome (ACS) remains a major global health burden, encompassing a spectrum of conditions from unstable angina to acute myocardial infarction. Despite advancements in early detection and management, ACS is often complicated by the development of heart failure. This systematic review and meta-analysis aimed to identify factors associated with the development of heart failure following acute coronary syndrome. A comprehensive search was conducted across PubMed, Embase, Cochrane Library, and Web of Science from January 2018 to November 2024. Studies evaluating clinical or biochemical predictors of heart failure development in adult patients with acute coronary syndrome were included. Out of the initially identified studies, nine studies met the inclusion criteria. The Newcastle-Ottawa Scale was used to assess the quality of included studies, with most studies demonstrating high quality. The pooled analysis revealed that older age, female sex, diabetes, hypertension, chronic obstructive pulmonary disease, atrial fibrillation, multivessel coronary disease, and reduced left ventricular ejection fraction were significant predictors of heart failure development following acute coronary syndrome. The presence of atrial fibrillation emerged as the strongest predictor, followed by reduced left ventricular ejection fraction and chronic obstructive pulmonary disease. While complete revascularization showed a protective trend, this association did not reach statistical significance. The findings were limited by the predominantly retrospective nature of included studies and heterogeneity in the assessment of certain risk factors. Future research should focus on prospective studies with larger cohorts and comprehensive evaluation of additional factors such as treatment delays and revascularization strategies. Understanding these predictors can facilitate early risk stratification and guide targeted interventions, potentially improving outcomes for patients with acute coronary syndrome.

## Introduction and background

Acute coronary syndrome (ACS) remains a major global health burden, encompassing a spectrum of conditions from unstable angina to acute myocardial infarction (AMI). Incidence rates range from 50 to 300 cases per 100,000 people per year, depending on the region and population studied [1,2]. Despite advancements in early detection and management, ACS is often complicated by the development of heart failure, a condition associated with significant morbidity, mortality, and healthcare costs [3]. Heart failure following ACS can result from a combination of left ventricular dysfunction, extensive myocardial damage, and adverse cardiac remodeling, highlighting the need to understand better the predictors of this devastating outcome [4]. 

The incidence of heart failure after ACS varies widely, influenced by factors such as patient demographics, comorbid conditions, and the extent of coronary artery disease [5]. Identifying patients at high risk of developing heart failure early in the course of ACS can enable targeted interventions, reduce the risk of heart failure progression, and improve overall clinical outcomes [6,7]. Traditional predictors such as left ventricular ejection fraction (LVEF), the extent of myocardial infarction (MI), and biomarkers like B-type natriuretic peptide (BNP) have been extensively studied [8]. However, contemporary research points to a more nuanced interplay of clinical, biochemical, and imaging markers. 

Recent studies have highlighted the importance of factors such as age, diabetes mellitus, renal dysfunction, and prior history of heart failure as strong predictors of heart failure in the ACS population [9]. Novel biomarkers, including high-sensitivity troponins, growth differentiation factor-15 (GDF-15), and soluble suppression of tumorigenesis-2 (sST2), have emerged as potential early indicators of increased heart failure risk [10]. Additionally, advanced imaging techniques like cardiac magnetic resonance imaging (MRI) provide detailed assessments of myocardial viability and fibrosis, offering insights beyond traditional echocardiographic parameters [10]. 

Despite these advances, there is considerable heterogeneity in the reported predictors of heart failure across studies, partly due to variations in study design, population characteristics, and definitions of heart failure [11-13]. Moreover, the role of sex, ethnicity, and genetic predispositions remains underexplored. This systematic review and meta-analysis aim to comprehensively synthesize the available evidence on the predictors of heart failure in patients with ACS, providing a consolidated framework to guide clinicians in risk stratification and personalized management strategies. 

In this context, understanding the diverse range of predictors, from conventional clinical variables to emerging biomarkers and imaging modalities, is critical for improving patient outcomes. By identifying consistent and robust predictors of heart failure in the ACS setting, this study seeks to inform future research directions and clinical guidelines, ultimately enhancing patient care and reducing the burden of heart failure post-ACS. 

## Review

Methodology 

Study Design 

This systematic review and meta-analysis were conducted following the guidelines outlined in the Preferred Reporting Items for Systematic Reviews and Meta-Analyses (PRISMA) statement. 

Information Sources and Search Strategy 

Two authors independently performed a comprehensive literature search across the following electronic databases: PubMed, Embase, Cochrane Library, and Web of Science. The search was conducted from 1 January 2018 to 5 November 2024, without language restrictions. Additionally, we screened the reference lists of relevant articles and reviews for any missed studies. The search strategy included terms related to "acute coronary syndrome," "myocardial infarction," "predictors," "risk factors," and "heart failure." Both Medical Subject Headings (MeSH) terms and free-text keywords were used to enhance the sensitivity of the search. Any disagreement between two authors during the process of searching was resolved through consensus and discussion with the principal investigator if required. 

Eligibility Criteria 

The eligibility criteria for this systematic review and meta-analysis included studies that enrolled adult patients (aged ≥18 years) diagnosed with ACS, encompassing unstable angina, non-ST-elevation myocardial infarction (NSTEMI), and ST-elevation myocardial infarction (STEMI). We included studies that evaluated clinical or biochemical predictors of heart failure development following an ACS event. Both observational studies (cohort and case-control) and randomized controlled trials (RCTs) were considered if they reported sufficient data on the incidence of heart failure or related adverse outcomes. We excluded studies that focused solely on pediatric populations or animal models or lacked specific outcome data on heart failure. Additionally, case reports, conference abstracts, and narrative reviews were excluded due to the limited data they provide. 

Study Selection 

All retrieved records were imported into reference management software, i.e., EndNote X9 (Clarivate, London, UK), and duplicates were removed. Two independent reviewers screened the titles and abstracts to identify potentially eligible studies. Full-text articles were then assessed for inclusion based on the predefined eligibility criteria. Disagreements were resolved by consensus or by consulting a third reviewer. 

Data Extraction 

Data extraction was carried out using a standardized and pre-piloted extraction form to ensure consistency and accuracy. For each included study, we collected details on study characteristics (author, publication year, country, study design, and sample size). We also extracted information about the predictors of heart failure, including adjusted and unadjusted effect measures. Outcome data focused on the incidence of heart failure, defined according to standardized clinical criteria, and follow-up duration. Two independent reviewers conducted the extraction, and discrepancies were resolved through discussion or consultation with a third reviewer to minimize errors and bias. 

Risk of Bias Assessment 

The risk of bias in the included studies was assessed using the Newcastle-Ottawa Scale (NOS) for cohort and case-control studies, evaluating domains such as selection bias, information bias, and confounding. Two reviewers independently performed the assessments, and discrepancies were resolved through discussion. Studies were categorized based on their NOS scores: more than 6 was considered good quality, 4-6 was considered fair quality, and less than 4 was considered low quality. This approach ensured a standardized and objective evaluation of study quality.

Statistical Analysis 

Data analysis was performed using RevMan Version 5.4.1 (Cochrane Collaboration, London, UK) and STATA Version 18.0 (StataCorp LLC, College Station, TX, USA). We conducted a meta-analysis of pooled effect estimates for predictors of heart failure using a random-effects model to account for heterogeneity among studies. Odds ratios (ORs) with 95% confidence intervals (CIs) were used as effect measures. A P-value less than 0.05 was considered significant. Heterogeneity was assessed using the I² statistic, with values >50% indicating substantial heterogeneity. We used a random-effect model despite of heterogeneity to deal with variation among the studies due to sample size, setting, population, and other causes. In this meta-analysis, we prioritized the use of adjusted measures for assessing the factors associated with heart failure. In case adjusted measures were not available, we used unadjusted measures. 

Results 

**Figure 1:**
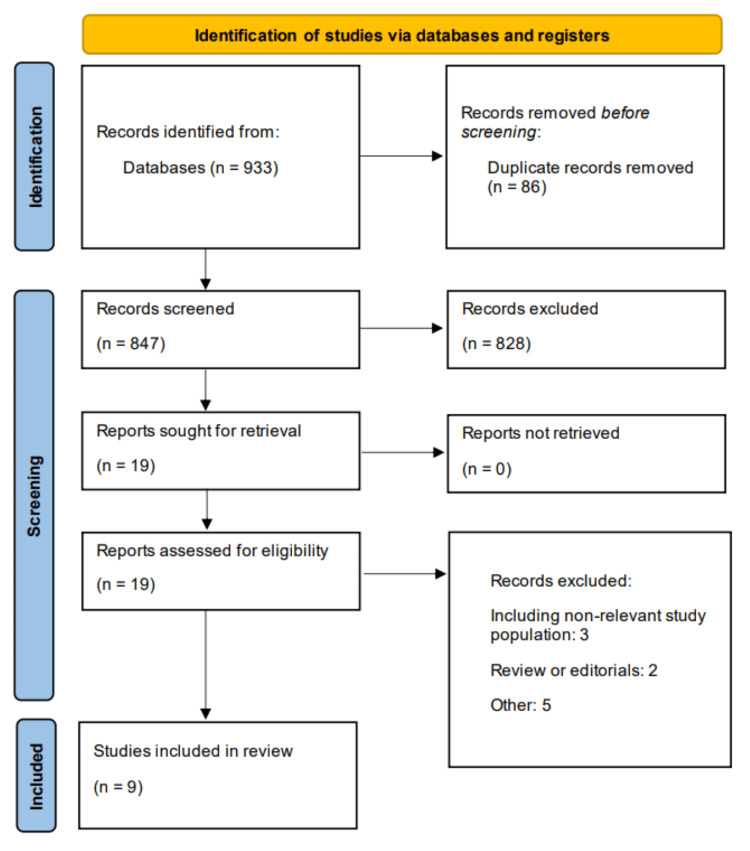
PRISMA flowchart of study selection. PRISMA: Preferred Reporting Items for Systematic Reviews and Meta-Analyses.

A total of 933 studies were initially identified through a comprehensive search of the electronic databases. After the removal of duplicates, 847 studies underwent title and abstract screening. Of these, 828 studies were excluded based on predefined eligibility criteria. The remaining 19 studies were selected for full-text review, after which 10 were excluded. Ultimately, nine studies met the inclusion criteria and were included in the final systematic review and meta-analysis. Figure 1 presents the PRISMA flowchart of study selection. Table 1 shows the characteristics of the included studies. Out of nine studies, eight were conducted retrospectively. Follow-up duration of included studies ranged from 20.1 to 52.8 months. Three studies were conducted in Portugal, three in Spain, and one in Morocco, Italy, and the Netherlands. Table 2 presents the quality assessment of the included studies. The majority of the studies were of high quality. Two studies are of fair quality, and none of the included studies are of low quality.

**Table 1:** Characteristics of included studies. NR: not reported.

Author ID	Year	Region	Study design	Follow-up	Sample size	Heart failure (n)
Bachar et al. [14]	2022	Morocco	Retrospective	NR	121	33
Bermejo et al. [15]	2018	Spain	Retrospective	52.8 Months	5064	1144
Cordero et al. [16]	2021	Spain	Prospective	63 Months	5962	567
Costa et al. [13]	2021	Portugal	Retrospective	43.6 Months	700	110
Filippo et al. [11]	2023	Italy	Retrospective	34.8 Months	14,699	1225
Lenselink et al. [17]	2024	Netherlands	Retrospective	44.4 Months	1172	121
Melendo-Viu et al. [18]	2019	Spain	Retrospective	20.1 Months	2158	59
Santos et al. [13]	2021	Portugal	Retrospective	NR	25,718	4003
Timoteo et al. [19]	2022	Portugal	Retrospective	NR	19,248	3330

**Table 2:** Assessment of included studies.

Author ID	Selection	Comparison	Assessment	Overall
Bachar et al. [14]	4	2	2	Good
Bermejo et al. [15]	3	2	3	Good
Cordero et al. [16]	3	2	2	Good
Costa et al. [13]	3	2	3	Good
Filippo et al. [11]	4	2	3	Good
Lenselink et al. [17]	4	2	2	Good
Melendo-Viu et al. [18]	3	2	3	Good
Santos et al. [13]	2	1	2	Fair
Timoteo et al. [19]	2	1	2	Fair

Incidence of Heart Failure Post-Acute Coronary Syndrome (ACS) 

**Figure 2:**
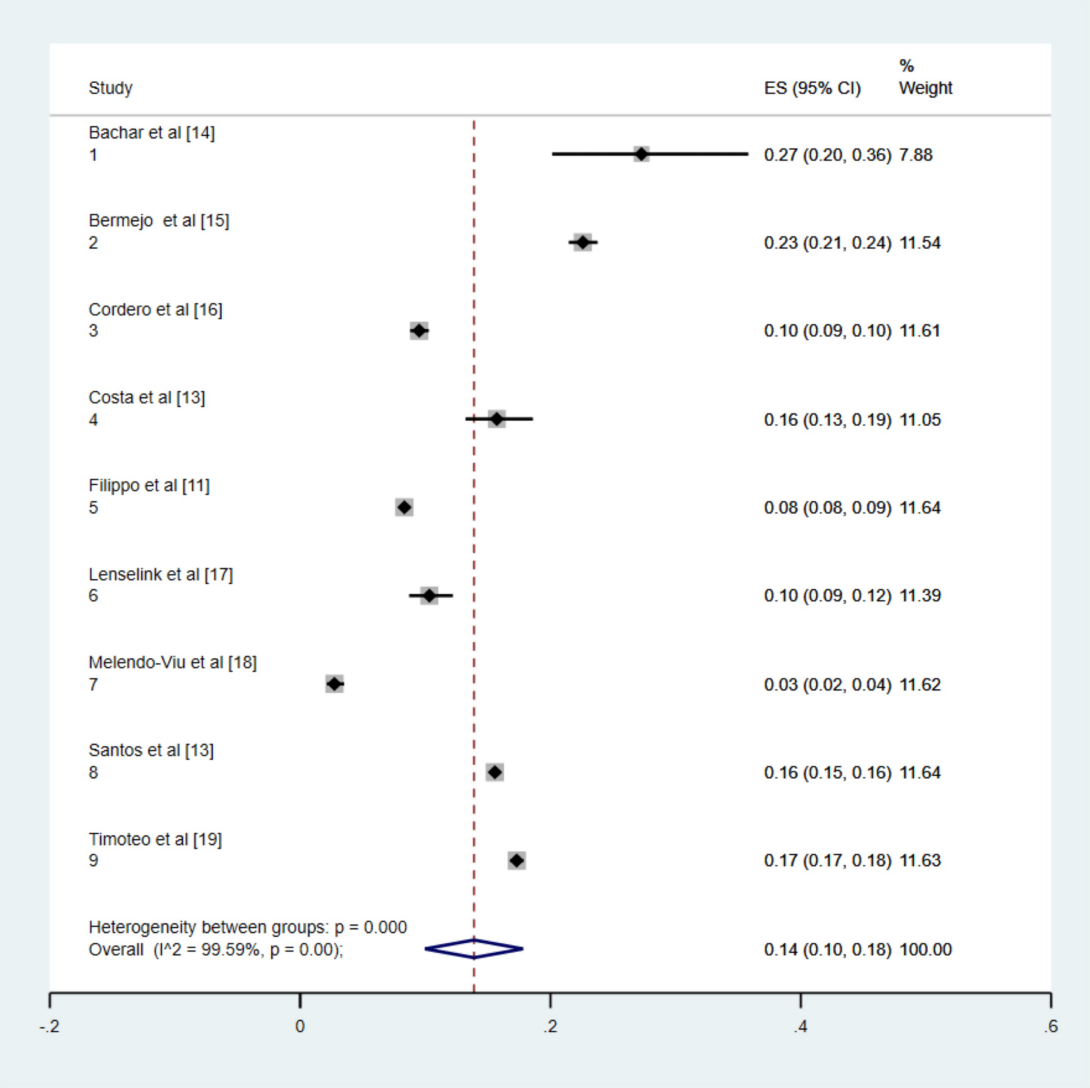
Forest plot presenting pooled incidence of heart failure post-ACS. ES: estimate, ACS: acute coronary syndrome. Note: This image is the author's own creation.

Figure 2 presents the forest plot illustrating the pooled incidence of heart failure following ACS. The overall pooled incidence of heart failure was estimated to be 13% (95% CI: 10%-17%). The incidence rates reported by individual studies varied widely, ranging from 3% to 27%. There was substantial heterogeneity among the study results (I² = 99%), likely attributed to variations in sample size, geographic regions, and differences in study populations. 

*Factors Associated With the Development of Heart Failure Post-Acute Coronary Syndrome​​​​​​​ (ACS)*​​​​​​​* *

Table 3 summarizes the factors associated with the development of heart failure following ACS. Older age was significantly linked to an increased risk of heart failure post-ACS (OR: 1.05, 95% CI: 1.01-1.09). Female sex was also found to be a significant risk factor for heart failure after ACS (OR: 1.21, 95% CI: 1.02-1.43). Additionally, patients with diabetes had a notably higher risk of developing heart failure (OR: 1.62, 95% CI: 1.48-1.77). The presence of hypertension was another significant predictor (OR: 1.51, 95% CI: 1.02-2.23). Chronic obstructive pulmonary disease (COPD) was strongly associated with an elevated risk of heart failure (OR: 1.82, 95% CI: 1.49-2.23). Furthermore, atrial fibrillation (AF) was a major risk factor, with affected patients exhibiting a significantly higher likelihood of developing heart failure (OR: 2.07, 95% CI: 1.62-2.65). Other significant predictors included multivessel coronary artery disease (OR: 1.52, 95% CI: 1.26-1.83) and LVEF below 50% (OR: 1.92, 95% CI: 1.37-2.71). Although patients who achieved complete revascularization had a lower risk of developing heart failure, this association was not statistically significant.

**Table 3:** Factors associated with heart failure post-ACS. OR: odds ratio; CI: confidence interval; COPD: chronic obstructive pulmonary disorder; LVEF: left ventricular ejection fraction; STEMI: ST elevated myocardial infarction; MVD: multivessel disease; ACS: acute coronary syndrome.

Factors	Number of studies	OR	95% CI	I-square
Age	5	1.05	1.01-1.09	85%
Gender (female)	8	1.21	1.02-1.43	59%
Diabetes	9	1.62	1.48-1.77	30%
Hypertension	5	1.51	1.02-2.23	90%
COPD	3	1.82	1.49-2.23	61%
Atrial fibrillation	5	2.07	1.62-2.65	47%
LVEF (<50%)	3	1.92	1.37-2.71	70%
STEMI	3	1.86	0.90-3.88	70%
MVD	4	1.52	1.26-1.83	0%
Complete vascularization	3	0.73	0.49-1.09	89%

Discussion 

This systematic and meta-analysis was conducted with the aim to determine the factors associated with the development of heart failure post-ACS. Some of the factors significantly associated with it included increased age, female gender, hypertension, diabetes, COPD, low LVEF, atrial fibrillation, and multivessel disease. 

The extensive adoption of percutaneous coronary intervention (PCI) has led to a decline in the number of patients experiencing ventricular systolic dysfunction after myocardial infarction [20]. The key predictor of heart failure during the follow-up period after an acute myocardial infarction (AMI) remains the presence of impaired ventricular systolic function. Patients who exhibit reduced LVEF following ACS are regarded as being at a significantly higher risk for developing heart failure [21]. LVEF reflects the percentage of blood ejected from the left ventricle with each heartbeat, serving as an important indicator of left ventricular systolic function. When LVEF is reduced, it signifies impaired heart contractility, often due to myocardial injury sustained during the ACS event [22]. The effect of low LVEF on the risk of developing heart failure was investigated in only three included studies. Given the crucial role of LVEF as a marker of left ventricular systolic function and its strong prognostic value in predicting adverse outcomes after ACS, the limited number of studies assessing this parameter highlights a significant gap in the literature. 

Diabetes and hypertension were significantly associated with the development of heart failure post-ACS. Similar findings were reported in the previous studies [23,24]. All studies reported these factors in the present meta-analysis reported a high risk of developing HF in patients with diabetes and hypertension. Diabetes induces myocardial changes, such as fibrosis and microvascular dysfunction, weakening the heart's resilience to ischemic injury [25]. Hypertension accelerates left ventricular hypertrophy and increases afterload, worsening myocardial stress during ACS. Both conditions exacerbate endothelial dysfunction and promote adverse cardiac remodeling, raising the risk of heart failure [26]. Recognizing and managing these factors is crucial for improving post-ACS outcomes. Another comorbidity reported in this meta-analysis associated with a high risk of heart failure is atrial fibrillation. All included studies reported a significant association between atrial fibrillation and heart failure. The irregular and rapid heart rhythm of AF disrupts coordinated atrial contraction, leading to reduced ventricular filling and decreased cardiac output, which can exacerbate existing myocardial dysfunction [27]. Additionally, AF increases myocardial oxygen demand and reduces coronary perfusion, further stressing the already compromised heart. These combined effects promote adverse cardiac remodeling and elevate the likelihood of heart failure development in ACS patients [28]. 

Our meta-analysis found that women with ACS face a higher risk of developing heart failure. Several factors may explain this increased risk in females. Women who experience myocardial infarction (MI) tend to be older than men and often have a higher burden of comorbidities, along with poorer functional status [29]. The effect of conditions like diabetes, hypertriglyceridemia, and metabolic syndrome on cardiovascular risk seems to be more pronounced in women than in men. Additionally, gender differences in the presentation of MI [30] and less aggressive treatment strategies for women, such as the underutilization of revascularization procedures [31,32], may further elevate the risk of heart failure in this population. Besides this, our analysis also reported that heart failure incidence increases with age. The occurrence of in-hospital heart failure is three times greater in patients aged 75-85 years compared to those aged 25-54 years. Following hospital discharge, the incidence of heart failure is six times higher in the older age group [33]. 

Several additional factors warrant further investigation in future studies, including the delay in treatment, which may contribute to worse outcomes in patients with ACS. The role of complete vascularization in reducing the risk of heart failure and improving long-term prognosis also needs more exploration. Additionally, the impact of different types of ACS (e.g., STEMI, NSTEMI, unstable angina) on heart failure risk should be better understood to tailor treatment strategies more effectively. 

Clinical and Research Implications

The findings of this systematic review and meta-analysis have important clinical and research implications. Clinically, recognizing key risk factors such as age, gender, diabetes, hypertension, low LVEF, atrial fibrillation, and multivessel disease can help identify ACS patients at high risk for developing heart failure, allowing for more targeted and timely interventions. Research-wise, further studies are needed to explore the role of delayed treatment, complete vascularization, and the specific impact of different ACS types on heart failure development. This will enhance understanding and inform more personalized treatment strategies, ultimately improving patient outcomes following ACS.

Study Limitations 

This systematic review and meta-analysis has several limitations. First, only nine studies were included, which may limit the generalizability of the findings. Additionally, the majority of the included studies were retrospective in nature, making them prone to inherent biases such as selection and recall bias. Some important factors, such as the impact of delayed treatment and complete vascularization, were not consistently assessed across all studies, potentially affecting the comprehensiveness of the analysis. Furthermore, the sample size of individual studies was often limited, which may reduce the statistical power of certain analyses. Given these limitations, further research with larger, prospective cohort studies is needed to validate these findings and address the gaps in the current evidence. Additionally, future studies should incorporate a more comprehensive set of risk factors, including treatment delays and revascularization strategies, to better understand their role in heart failure development post-ACS. 

## Conclusions

This systematic review and meta-analysis identified several significant predictors of heart failure following acute coronary syndrome. The findings revealed that older age, female sex, diabetes, hypertension, COPD, atrial fibrillation, multivessel coronary disease, and reduced left ventricular ejection fraction are important risk factors for developing heart failure after ACS. Understanding these predictors is crucial for early risk stratification and targeted interventions in ACS patients. However, the analysis was limited by the small number of included studies, their retrospective nature, and variable assessment of important factors like treatment delays and revascularization strategies. Future research should focus on prospective studies with larger cohorts and comprehensive risk factor assessment to validate these findings, ultimately leading to improved risk prediction models and better outcomes for patients with ACS.
